# Proliferative verrucous leukoplakia management requires x-ray surveillance. A retrospective study of 78 cases

**DOI:** 10.1007/s00784-026-06815-w

**Published:** 2026-03-19

**Authors:** Nikoleta Molnarova, Veronika Liskova, Tomas Malkus, Petra Hauerova, Pavel Andrle, Lukas Hauer, Ondrej Topolcan, Jan Liska

**Affiliations:** 1 Faculty of Medicine in Pilsen, Charles University, Alej Svobody 1655/76, Pilsen, 32300 Czechia; 2 Dentistry Clinic, University Hospital Pilsen, Alej Svobody 80, Pilsen, 30460 Czechia; 3 Department of Imaging methods, University Hospital Pilsen, Alej Svobody 80, Pilsen, 30460 Czechia; 4 Central Laboratory of Immuno-analysis, University Hospital, Ed. Beneše 13, Pilsen, 30599 Czechia; 5 Biobank of Faculty of Medicine in Pilsen, University Hospital Pilsen, Ed. Beneše 13, Pilsen, 30599 Czechia

**Keywords:** Proliferative Verrucous Leukoplakia, Malignant Transformation, Oral Squamous Cell Carcinoma, Alveolar Bone Resorption, Cone Beam Computed Tomography

## Abstract

**Background:**

Proliferative verrucous leukoplakia (PVL) is a very rare lesion of the oral mucosa with frequent development of oral epithelial dysplasia and a very high ratio of malignant transformation (MT). The aim of this study is to correlate radiographic assessment of alveolar bone resorption with the clinical manifestations and course of PVL in order to improve therapy.

**Materials and methods:**

The study evaluates the resorptive changes on alveolar ridges in 78 cases of PVL, confirmed by clinical and pathological criteria. All patients were treated at the Oral Medicine Department, Dentistry Clinic, University Hospital Pilsen. Each case was examined for bone defects using imaging methods such as X-ray intraoral scans, orthopantomograms, or cone beam computed tomography (CBCT). The location and extent of the resorption were correlated with the distribution of PVL lesions in the oral cavity and the course of the mucosal disease. Cases with MT underwent whole-body examination using hybrid imaging techniques, including positron emission tomography (PET) combined with either magnetic resonance imaging (MRI) or computed tomography (CT).

**Results:**

A correlation between the alveolar resorptive process and the development of PVL lesions was found in 66.7% of PVL cases (52/78). In one-sixth of cases (13/78), progressive bone loss paralleled worsening mucosal status and increased tooth mobility. Radiographic follow-ups delineated areas indicated for antimicrobial and anti-inflammatory therapy to address aggressive local cofactors in PVL. Oral squamous cell carcinoma developed in 37% of cases (29/78). Combined clinical and radiologic surveillance enabled early detection of malignant transformation (MT) in 5 cases (17% of MTs).

**Conclusion:**

Routine radiologic assessment is an essential component of PVL management. Radiography and CT improve detection of local cofactors, help define the scope of therapy, and may indicate the onset of MT before overt clinical manifestations.

**Supplementary Information:**

The online version contains supplementary material available at 10.1007/s00784-026-06815-w.

## Introduction

Proliferative verrucous leukoplakia (PVL) is a very aggressive and relatively rare form of oral leukoplakia [1]. It was first described by Hansen et al. in 1985 [2]. PVL typically begins as a white plaque and eventually becomes a multifocal, slow-growing lesion that is resistant to treatment, including surgery. Recurrence is common, and malignant transformation (MT) is frequent [2–4]. According to recent meta-analyses, the MT rate in PVL ranges from 44% to 100% [5, 6]. The most commonly affected areas are the alveolar ridge, tongue and buccal mucosa; the palate or floor of the mouth may also be involved [7, 8].

The World Health Organization (WHO) initially classified PVL as “a precancerous lesion”. In 2007, experts recommended replacing ‘precancerous’ with ‘potentially malignant oral disorders’ [7, 9–12]. The latter was recommended because it recognizes that not all such disorders progress to oral squamous cell carcinoma (OSCC) [7–12].

The histopathological profile of PVL typically includes hyperkeratosis, hyperplasia, acanthosis, and frequently a lichenoid lymphocytic infiltrate adjacent to the basal membrane [13, 14]. Dysplasia may be absent in the early stages. As PVL progresses clinically, severe epithelial dysplasia may develop, potentially advancing to verrucous carcinoma or OSCC [6, 15, 16]. Diagnostic criteria for PVL have been proposed by different research teams [17–19]. In 2010, Prof. Cerero-Lapiedra and colleagues classified the criteria into five major and four minor categories [18, 20, 21].

PVL management requires frequent follow-ups, including repeat biopsies due to clinical progression and lesion recurrence [8, 13, 15, 22]. Even patients without recurrence should be examined at least twice a year [15]. The course of PVL can be influenced by bacterial inflammation in the gingiva and periodontal tissues. This pathological cofactor is associated with aggressive periodontal pathogens [23]. Oral bacteria are known to cause local inflammation and are also frequently linked to oral and distant cancers [24, 25].

**Fig. 1:**
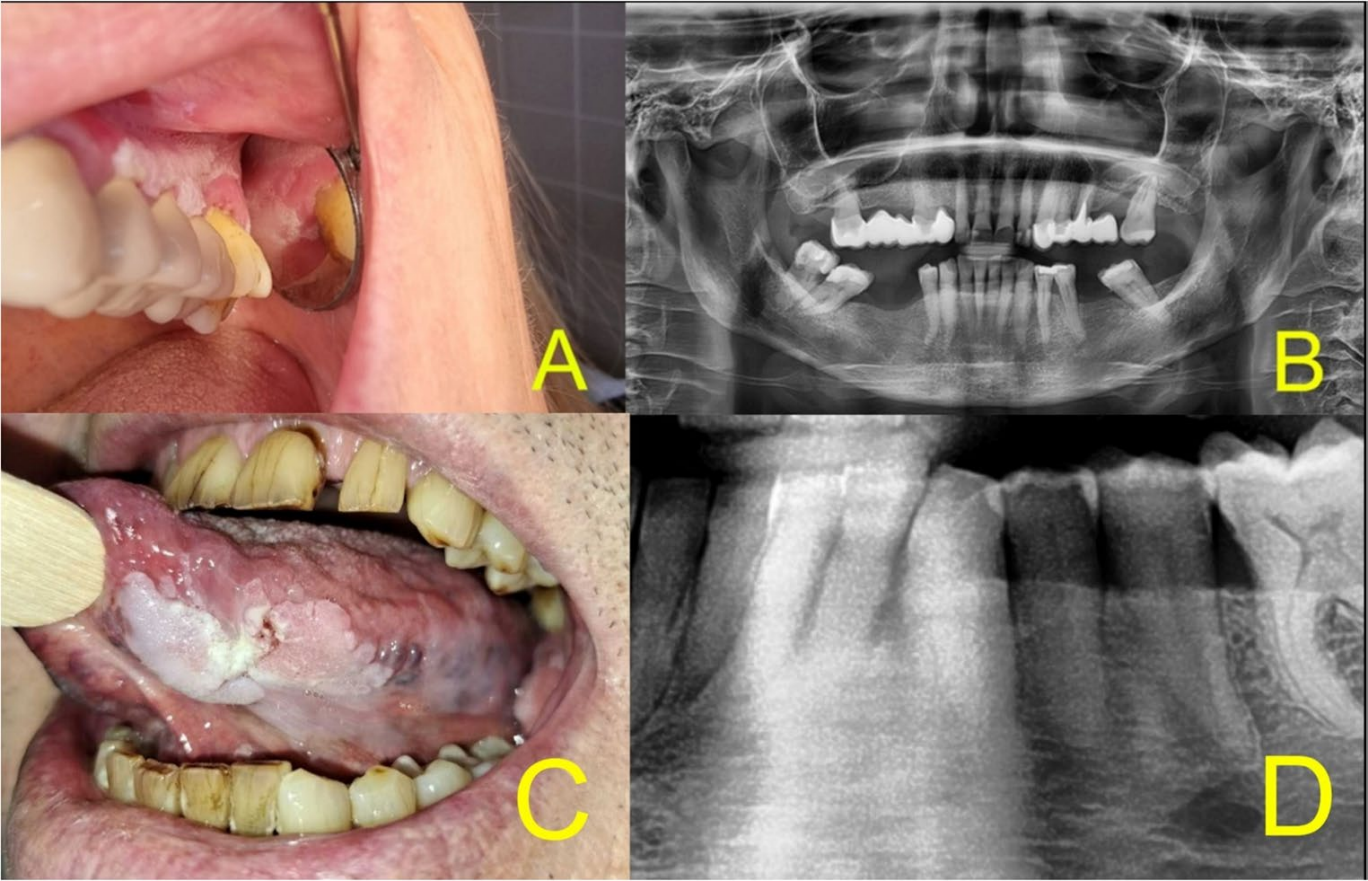
Correlation of mucosal lesions and resorptive defects in PVL (A + B, C + D); (**A**) the warty surface of verrucous leukoplakia characteristic for PVL on alveolar mucosa and gingiva in localization 24–27 (female, 67 years old). (**B**) OPG showing directly correlated alveolar bone resorption in the area of PVL, mesial to tooth 27. (**C**) Large PVL plaque on the left lateral border of the tongue (male, 70 years old). (**D**) OPG of the lower anterior region showing indirectly correlated bone resorption near tooth 33. Abbreviations in Fig. 1: OPG = orthopantomogram, PVL = proliferative verrucous leukoplakia.

As part of the clinical management of oral epithelial lesions, radiological examination is performed only sporadically. Dentists are generally familiar with resorptive changes in the alveolar bone caused by periodontitis. To detect these changes, intraoral radiography—such as radiovisiography (RVG)—and extraoral imaging modalities, including orthopantomography (OPG) and cone-beam computed tomography (CBCT), are commonly used. The correlation between alveolar PVL and adjacent alveolar bone resorption is shown in Fig. 1.

In PVL, it is essential to differentiate between typical periodontal bone resorption and bone defects caused by the development of OSCC. In cases of MT, it is necessary to determine both the histological grade and the clinical stage [24, 26]. This was traditionally assessed using computed tomography (CT) scans of the head and neck. Currently, hybrid imaging techniques such as positron emission tomography (PET) combined with CT or magnetic resonance imaging (MRI), are preferred.

The aim of this study is to highlight the importance of correlating oral manifestations of PVL with radiological examination of the alveolar bone. The current literature does not adequately address this topic, despite the high risk of malignant transformation associated with PVL.

## Materials and methods

Between 1994 and 2025, 78 cases of PVL were diagnosed and treated at the Department of Oral Medicine, Dentistry Clinic, University Hospital Pilsen.

### Inclusion criteria

PVL cases verified according to the clinicopathological criteria of Cerero-Lapiedra. Only cases meeting either three major criteria, or two major plus at least two minor criteria, were included. Furthermore, only PVL patients who had undergone both clinical periodontal assessment and radiological examination of the alveolar bones and teeth were considered.

### Exclusion criteria

Clinical manifestations mimicking PVL without biopsy confirmation of diagnosis. Verified PVL cases without clinical periodontal and dental radiological examination were also excluded.

The diagnosis of PVL was established by strict adherence to the predefined inclusion criteria. Cases that merely resembled PVL (oral lichen planus, other types of leukoplakia) were excluded by the authors prior to evaluation of the radiographic documentation.

Two PVL-positive patients were excluded due to the absence of dental X-rays: one case of PVL with labial MT in a 78-year-old female, and one case of PVL without MT in a 60-year-old male. The diagnostic criteria for PVL according to Cerero-Lapiedra are summarized in Table 1.

**Table 1:** Cerero-Lapiedra et. al criteria for PVL

Major criteria	Minor criteria
A leukoplakia lesion with more than two different oral sites, which is most frequently found in the gingiva, alveolar processes and palate	Oral leukoplakia with a total lesion area of at least 3 cm2
The existence of a verrucous area	The patient is female
The lesions have spread during the development of the disease	The patient is a nonsmoker
There has been a recurrence in a previously treated area	Disease evolution is higher than 5 years
Histopathological profile varies from simple epithelial hyperkeratosis to verrucous hyperplasia, verrucous carcinoma or oral squamous cell carcinoma in situ or infiltrating	

**Fig. 2:**
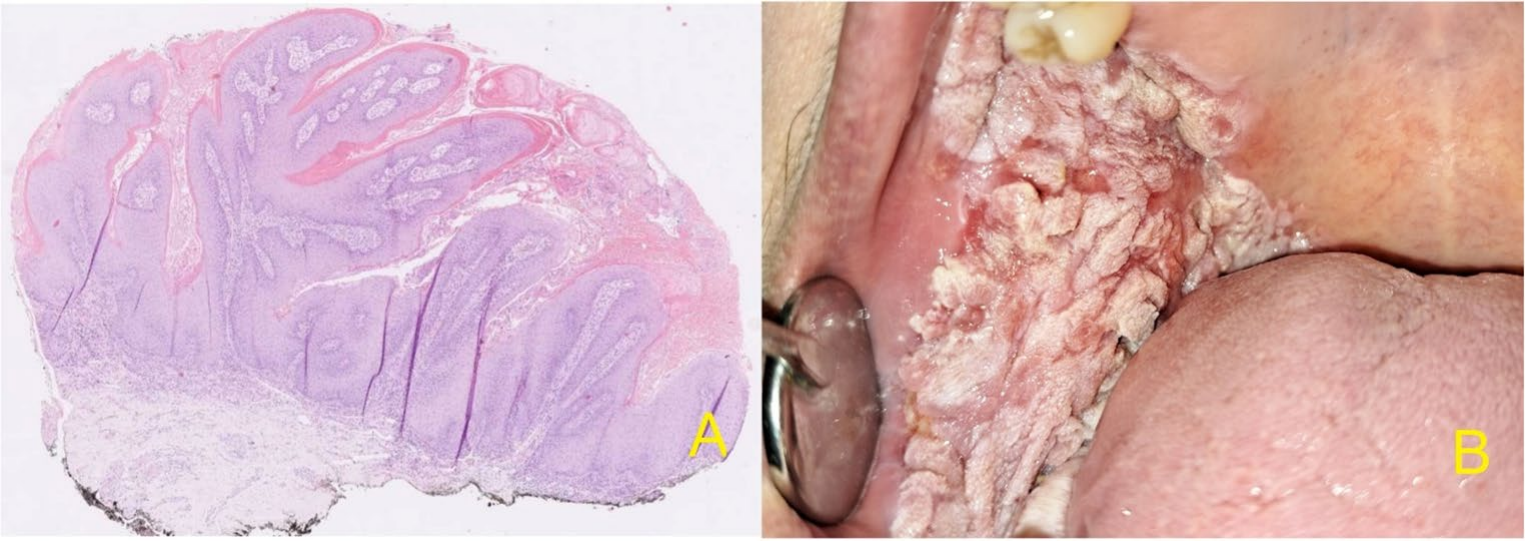
Correlation between histological-clinical aspects in PVL (female, 62 years). Malignant transformation in this case was treated by local radical excision. (**A**) Biopsy demonstrating extensive hyperplasia, hyperkeratosis, and acanthosis (Archive of Professor Kacerovska, Sikl´s Department of Pathology, University Hospital Pilsen). (**B**) Papillomatous, non-homogeneous lesions involving the entire right buccal mucosa with extension to both alveolar processes. Abbreviations in Fig. 2: PVL = proliferative verrucous leukoplakia

Figure 2 illustrates the histological–clinical correlation of a PVL lesion in the buccal and alveolar mucosa. 

A medical history was obtained, including information on nicotine use. The distribution of PVL lesions was evaluated. In dentate patients, the status of periodontal tissues was examined using clinical probing with a round-tip WHO probe. If periodontitis was diagnosed, cases were classified according to either the 1999 criteria (chronic or aggressive periodontitis) or the 2017 criteria (Staging I–IV and Grading A–C).

In cases where a true periodontal pocket (> 3.5 mm) was detected, a test for aggressive periodontal pathogens was performed. Subgingival plaque was collected with sterile paper points and analyzed using a DNA test (VariOr-Dento Plus system, Gen-Trend Co.), which can detect 12 bacterial species with known aggressive behavior in periodontal tissue and quantify their levels in plaque. Based on the results of periodontal pathogen testing and clinical examination of the periodontium, the risk of periodontitis progression was graded on a scale from 0 to 3.

Tooth mobility was routinely assessed by palpation (Grade 0–3, ranging from no movement to excessive horizontal and vertical mobility in the alveolar socket). Because the etiology of PVL remains unknown, anamnestic data collection was limited to smoking status.

Radiological screening was performed using OPG or CBCT. When necessary, OPG images were further refined with additional RVG. Bone resorption was defined as loss of compact bone accompanied by an increase of more than 1 mm in the distance between the cementoenamel junction and the alveolar bone. In edentulous sections of the jaws, the extent of the defect with loss of compact bone was measured. The radiographic imaging software used for these assessments enabled measurements with a precision of 0.1 mm (Medicalc4, Medicalc Software Co., Czechia). To minimize inter-reviewer variability, all images were evaluated by a single periodontist. The correlation between the distribution of mucosal lesions and alveolar resorption was assessed retrospectively. Alveolar resorption was classified as direct when localized at the site of clinically evident PVL and as indirect when located in areas adjacent to PVL lesions. During follow-up, radiological examination was repeated in cases of clinical progression of lesions or deterioration of the periodontal condition in the affected area. Patients with a risk of MT were examined using hybrid imaging methods (PET-CT/MR).

## Results

The average follow-up period was 6.87 years. The male-to-female ratio was 1:1.5 (*n* = 31:47; 60.3% female). The average age of patients at the time of the study was 66.2 years. No anamnestic predisposition for the development of PVL was identified; and 43% of patients were non-smokers (34/78). Periodontal evaluation, including assessment for periodontal pathogens, was performed in 62 dentate patients with PVL. The mean risk score for periodontitis progression was 2.66 on a 0–3 scale. Clinical follow-up was performed twice a year in stable cases and every two months in high-risk cases. In total, 136 PVL lesions were monitored in 78 patients, without distinction between mandibular and maxillary localization. PVL recurred in 65% of patients (51/78). MT was diagnosed in 37% of patients (29/78), in whom 39 OSCCs developed. The counts and percentages of PVL lesions and OSCCs are presented in Table 2. OSCC recurrence was observed in 27.6% of patients (8/29). During the follow-up period, seven patients died from OSCC-related complications.

**Table 2:** Localization of PVL lesions and OSCCs

Localization	PVL	% of 136	OSCC	% of 39
Alveolar ridge	51	37.5	18	46.2
Tongue	33	24.3	8	20.5
Buccal Mucosa	28	20.6	8	20.5
Floor of the Mouth	13	9.6	1	2.5
Labial Mucosa	6	4.4	2	5.1
Palatal Mucosa	5	3.7	2	5.1

*PVL* proliferative verrucous leukoplakia, *OSCC* oral squamous cell carcinoma

Radiological assessment most frequently involved OPG, performed in 91% of PVL cases (71/78), followed by CBCT in 27% (21/78). RVG was additionally used in 17% (13/78). Only patients with MT underwent CT of the head and neck (10/29) or hybrid imaging, with PET/CT (22/29) being more common than PET/MR (6/29). A positive correlation between alveolar bone resorption (on X-ray) and clinical manifestation of PVL was identified in 66.7% of cases (52/78). Direct correlation (alveolar resorption adjacent to alveolar PVL) was observed in 42.3% (33/78). Progressive periodontal resorption in the vicinity of “contact” non-alveolar PVL lesions was found in 24.4% (19/78). These indirect correlations were most frequent with tongue lesions 68.4% (13/19), less often on the floor of the mouth 15.8%, buccal mucosa 10.5%, and labial mucosa 5.3% (*n* = 3, 2, 1 out of 19). Assessed resorptions were consistently associated with probing depths > 5.5 mm and increased levels of periodontal pathogens.

**Fig. 3:**
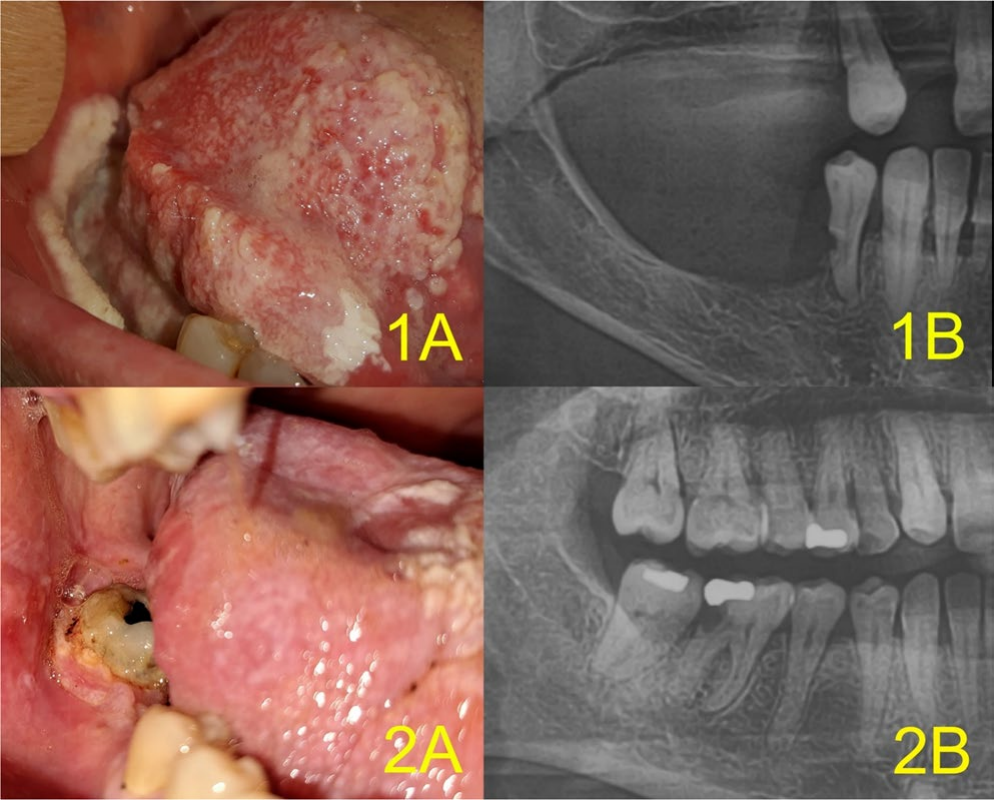
Clinical-radiological correlation indicating MT of two PVL: Case 1 and 2. 1**A**) MT of lesion involving the right buccal mucosa, mandibular alveolar ridge, floor of the mouth, and tongue margin (female, 68 years). 1**B**) OPG showing mandibular resorption distal to tooth 44. 2 **A**) Verrucous carcinoma arising from PVL at tooth 47 (female, 45 years). 2**B**) Resorption mesial to tooth 47; tooth 46 had been extracted shortly before biopsy due to caries destruction. Abbreviations in Fig. 3: MT = malignant transformation, PVL = proliferative verrucous leukoplakia, OPG =orthopantomogram

**Fig. 4:**
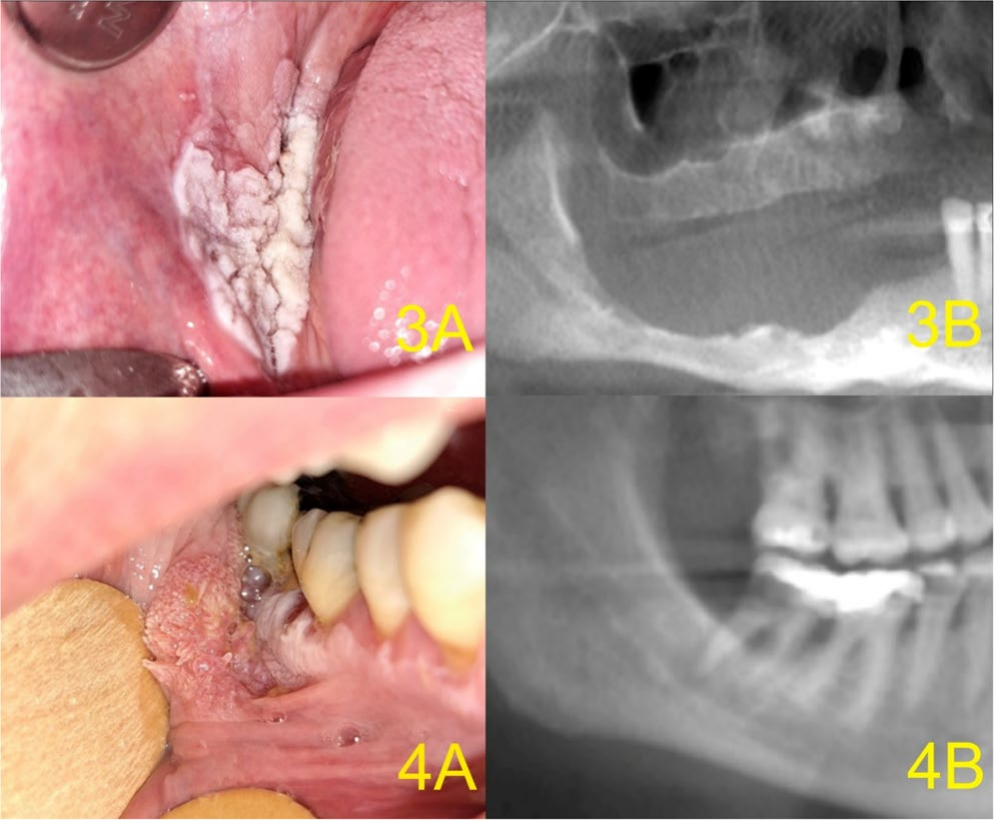
Clinical–radiological correlation of MT in PVL (Cases 3 and 4). 3**A**) MT of the right buccal mucosa and mandibular alveolar ridge (male, 67 years). 3**B**) OPG with mandibular resorption in the molar region. 4A) Verrucous carcinoma of the buccal mucosa with adjacent PVL in the 47–44 region (female, 70 years). 4**B**) Resorption in the furcation of tooth 47 and adjacent bone. Abbreviations in Fig. 4: MT = malignant transformation, PVL = proliferative verrucous leukoplakia, OPG =orthopantomogram

Periodic periodontal re-measurement and radiographic follow-up were performed in cases of increasing tooth mobility. The extent of alveolar resorption was regularly monitored. The radiological protocol included preventive OPG performed once yearly, in accordance with the periodontal recall protocol. When suspicious findings were present, tooth extraction and re-excision with extensive excochleation of the socket were indicated. Frequent evaluation of jaw bone defects facilitated early identification of MT in five cases of alveolar PVL (17% of 29 MT cases). In these cases, re-excision was indicated based on the detection of an alveolar defect despite clinical stability and a recent biopsy showing no evidence of malignancy. Four representative cases with clinical–radiological correlation are presented in Figs. 3 and 4 (the fifth case was previously published in our review article on PVL) [27]. In these cases, perilesional teeth were tested for mobility. Mobility increased from Grade 0 to Grade 2 in three of five patients, while the remaining two lesions occurred in edentulous regions. Three patients were diagnosed with moderate chronic periodontitis (Stage III, Grade B). Two patients were suspected of having aggressive periodontitis associated with *Aggregatibacter actinomycetemcomitans* (*A.a.*) or Stage IV, Grade C periodontitis. DNA tests for periodontal pathogens were performed in 4 of these 5 cases. DNA tests for periodontal pathogens were performed in 4 of these 5 cases. *A.a.* was confirmed in 2 cases, and in 3 of 4 cases increased volumes of the “red and orange complex” of aggressive periodontal pathogens were detected.

## Discussion

Our study highlights the necessity of radiological monitoring of aggressive mucosal lesions in the oral, particularly in the alveolar region. To the best of our knowledge, this is the first study to provide a comprehensive clinical–radiological correlation of PVL cases. Radiographic examination plays a crucial role in PVL management by guiding decisions on re-biopsy and radical surgery. 

PVL is among the most dangerous types of potentially malignant oral disorders. The clinical–pathological correlation of PVL is challenging, particularly in the early stages of the disease [5]. Clinically, PVL initially presents as homogeneous leukoplakia. As the disease progresses, the distribution of lesions on the buccal and alveolar mucosa may resemble oral lichen planus. Individual, maturing verrucous lesions can give the impression of human papillomavirus infection [5, 14]. Histopathological findings often include Candida hyphae and a lichenoid lymphocytic infiltrate and may lead the pathologist to interpret the condition as the less aggressive chronic hyperplastic candidiasis or, alternatively, oral lichen planus [13, 14]. Despite extensive efforts, purely histopathological criteria that reliably identify PVL have not yet been established [15]. For these reasons, close clinicopathological correlation is essential for the diagnosis of PVL. Management remains difficult owing to the absence of established etiological factors [2, 3, 28]. Consequently, therapeutic decisions often rely on careful observation or on suspected cofactors that may modify disease progression.

Although PVL was previously linked to smoking, this association has been refuted in recent studies [2, 8, 13, 22, 29]. Almost half of the patients in our study were non-smokers (43% at 34/78). Historically, numerous therapeutic approaches—including surgery, laser therapy, and radiotherapy—have been attempted, but with limited success [13, 15, 30–34].

To date, no studies have specifically applied diagnostic dental radiology for the direct follow-up of OPMDs. When gingival status is assessed, the periodontal condition is typically evaluated only through clinical examination with standardized indices, without radiographic assessment [35, 36]. For lesions located on the alveolar mucosa, the addition of X-ray imaging is meaningful. In some cases, the activity of hyperkeratotic mucosal lesions can only be monitored by their extent and degree of hyperplasia. In both dentate and edentulous patients, the surface of the compact alveolar bone may be evaluated radiologically. Bone resorption can alert clinicians to a high-risk development. Radiographic diagnostics are particularly valuable in PVL, enabling early detection of disease progression or MT of alveolar lesions. In such situations, the examining clinician may be discouraged from performing re-biopsies to confirm malignant transformation because of the apparent clinical stability of the lesions, and communication with the patient regarding additional surgical procedures may be complicated by a relatively recent biopsy with non-malignant findings.

Gingiva and alveolar mucosa were the most frequent sites of PVL in our study, consistent with previous publications [15, 20, 30, 37, 38]. While other studies described alveolar PVL in up to 87% of cases, our cohort showed a lower frequency of 37.5% [8, 12]. PVL progression from a single interdental space to extensive collateral involvement may be linked to inflammatory processes in periodontal tissue [2, 23]. Chronic or aggressive periodontal inflammation likely acts as a cofactor, driving PVL progression in both alveolar lesions and adjacent “contact” lesions on the buccal mucosa or tongue [23]. There was clear evidence that aggressive gingival inflammation influenced the progression of contact mucosal lesions of PVL, most frequently observed on the tongue (13 of 19 cases). Our study supports the concept that aggressive inflammation contributes to the progression of PVL lesions, as demonstrated by the high correlation between mucosal manifestations and alveolar bone resorption. This correlation was observed in 66.7% of cases, with direct involvement confirmed in 42.3% and indirect involvement in 24.4%. Such an association is important when considering therapeutic guidelines, as it highlights a potential target for reducing disease progression. Antimicrobial therapy combined with periodontal treatment and surgical procedures may improve outcomes in PVL management.

In cases of an increased burden of aggressive periodontal pathogens, the use of adjunctive antibiotics or laser-assisted periodontal decontamination may be considered. In our view, limiting chronic inflammation may help eliminate one of the cofactors involved in OPMD progression. In patients with PVL lesions, and in contrast to conventional periodontal therapy, we favor tooth extraction with removal of the surrounding tissue for biopsy when periodontal pockets exceed 6 mm and are associated with tooth mobility.

Adjunctive antimicrobial therapy, in combination with periodontal treatment and surgical procedures, may lead to more successful outcomes in the management of PVL. The involvement of periodontal pathogens in cancer development, both locally and at distant sites, is well established in recent studies [39–43]. Three main mechanisms have been described for their role in carcinogenesis: (1) bacterial stimulation of chronic inflammation, (2) promotion of cell proliferation through NF-κB activation and inhibition of apoptosis, and (3) production of toxic substances that act as procarcinogens [25, 44, 45]. The progression of OPMD to OSCC is influenced by the activity of multiple cytokines, including interleukins 1, 6, and 8 [3, 46]. NF-κB activation upregulates the expression and subsequent activity of interleukin 1-beta (IL-1β). IL-1β is a potent pro-inflammatory cytokine with a dual role in disease development, as its pathological effect promotes tumor growth and progression [46].

Optimized therapy for PVL remains elusive. Outcomes in our cohort were slightly better but still unsatisfactory, with a recurrence rate of 65% (51/78) compared to 71.2–100% in the literature [8, 47]. A similar pattern was observed for MT, with 37% in our cohort versus 40–100% in published studies [7, 8, 14, 29, 30, 48–51]. These improved outcomes may be attributed to comprehensive PVL management, including frequent follow-ups, monitoring of periodontal status, and subsequent antimicrobial and surgical interventions [8, 13, 22]. As previously reported, the alveolar region was the most frequent site of MT [52, 53], and this was supported by our findings, with 46.2% of OSCC cases arising from alveolar PVL. Unfortunately, overall mortality from OSCC originating in PVL remains high, with 24.1% in our study (7/29) compared to 21.29% reported previously [49, 53].

One limitation of this study is its single-center design. Given the rarity of PVL, assembling larger cohorts is challenging. Future multicenter studies will be essential to improve understanding and prognosis of this disease.

## Conclusion

Our findings support regular radiological examination as a useful adjunct tool for identifying higher-risk sites in PVL management. Radiographic and CT imaging enhances risk detection, help guide surgical management, and may allow earlier identification of malignant transformation than clinical examination alone.

## Supplementary Information

Below is the link to the electronic supplementary material.

Supplementary Material 1 (XLSX 16.3 KB)

## Data Availability

The raw data for the dataset of the study on the development of PVL correlated to radiographic examination are in the supplementary file.
